# Vascular Microenvironment, Tumor Immunity and Immunotherapy

**DOI:** 10.3389/fimmu.2021.811485

**Published:** 2021-12-20

**Authors:** Zachary Lamplugh, Yi Fan

**Affiliations:** Department of Radiation Oncology, University of Pennsylvania, Philadelphia, PA, United States

**Keywords:** immunity, immunotherapy, tumor microenvironment, endothelial cells, immune suppression, T cells, infiltration, exhaustion

## Abstract

Immunotherapy holds great promise for treating cancer. Nonetheless, T cell-based immunotherapy of solid tumors has remained challenging, largely due to the lack of universal tumor-specific antigens and an immunosuppressive tumor microenvironment (TME) that inhibits lymphocyte infiltration and activation. Aberrant vascularity characterizes malignant solid tumors, which fuels the formation of an immune-hostile microenvironment and induces tumor resistance to immunotherapy, emerging as a crucial target for adjuvant treatment in cancer immunotherapy. In this review, we discuss the molecular and cellular basis of vascular microenvironment-mediated tumor evasion of immune responses and resistance to immunotherapy, with a focus on vessel abnormality, dysfunctional adhesion, immunosuppressive niche, and microenvironmental stress in tumor vasculature. We provide an overview of opportunities and challenges related to these mechanisms. We also propose genetic programming of tumor endothelial cells as an alternative approach to recondition the vascular microenvironment and to overcome tumor resistance to immunotherapy.

## Introduction

Tumor vasculature has presented a complex problem to achieving therapeutic success across all cancer treatment modalities. Solid tumors exhibit aberrant vasculature composed of tumor endothelial cells (ECs) that present a physical barrier to treatment as well as promote aggressive tumor phenotypes that are prone to become aggressive and metastatic. The abnormal blood circulation leads to insufficient perfusion and drug delivery and creates pockets of hypoxia, which enhances tumor heterogeneity and promotes resistance to cancer therapies such as chemotherapy, radiation, and immunotherapy. Growing evidence shows that tumor ECs drive immunosuppression through selective immune cell recruitment, metabolic competition and epigenetic remodeling, suggesting the vascular microenvironment as an emerging target for solid tumor immunotherapy. Of various blood vessel-targeting treatments, anti-angiogenic therapy that primarily blocks vascular endothelial growth factor (VEGF) pathway has been widely pursued to inhibit tumor vascularization to starve cancer of oxygen and nutrients or to normalize tumor vasculature to overcome hypoxia-mediated treatment resistance. Combination of anti-angiogenic or other next-generation vasculature-targeting therapies with immunotherapy may, therefore, offer exciting opportunities for treating solid tumors. In this review we look to highlight the challenges implicated in immunotherapy for solid tumors, the landscape of the vascular microenvironment, and examine how these unique obstacles presented by tumor ECs impact current treatment options.

## T Cell-Based Immunotherapy of Solid Tumors

### Current Approaches

The promise of immunotherapy as an approach to solid tumors offers a potentially significant upgrade over traditional treatments such as chemotherapy, radiation, and molecular targeted therapy. While the therapeutic windows of chemotherapy and radiation are limited to when the drug is being applied or is present inside the body, immunotherapy has the capacity to remain active for an extended period once the immune system has been modified to target cancer cells. Immune checkpoint blockade and chimeric antigen receptor T cell (CAR-T) therapy remain the two dominant approaches for current T cell-based immunotherapy. Immune checkpoint blockade sought to remove the “brakes” or inhibitory checkpoints that prevented our immune systems from reaching their full potential as a natural defense against cancer. Monoclonal antibodies targeting immune cell expressed cytotoxic T lymphocyte-associated protein 4 (CTLA-4) and programmed-death 1 (PD-1) along with TME expressed programmed death-ligand 1 (PD-L1) have led to approved treatment options for solid tumors such as melanoma, non-small cell lung cancer (NSCLC), and urothelial carcinoma (1). Although approved treatments exist, many factors play a role in whether immune checkpoint blockade will be successful such as age, sex, gene expression, mutation burden, epigenetic and metabolic alternation in tumor cells, and stage of disease leaving a lot to be desired in effectiveness of the treatment (2). The notion of exploiting antigen fragment recognition of T cells by introducing genetic material that encodes for antibody recognition was first proposed by Gross et al. in 1989 (3). After development of several generations of CAR constructs, the US Food and Drug Administration (FDA) has recently approved drugs developed using this method such as Novartis’s Kymriah and Kite Pharma’s Yescarta that target tumor-associated antigens (TAAs) in hematopoietic malignancies (4–6). As of now there are no FDA approved CAR-T cell treatments available for solid tumors as there are many challenges unique to solid tumors that make CAR-T cells challenging to implement.

### Therapeutic Challenges

The first major challenge that exist for implementing CAR-T cell therapy for solid tumors is the absence of universal tumor-specific antigens (TSAs) or TAAs in the cancer of interest. Ideally the antigen should be expressed selectively on tumor cells or at least highly expressed on tumor cells while being minimally expressed on healthy tissues. The majority of current CAR-T cell target antigens exist on healthy tissues leading to “on-target/off-tumor” toxicity (7, 8). A second important characteristic of the antigen is that it should be expressed on all cancerous cells. If any cells within the tumor do not express the target antigen, then the chances of a treatment having a meaningful impact decrease significantly. Even if the target antigen is present on all of the tumor cells, loss of antigenicity can still occur through immune escape mechanisms such as loss of antigen presentation or mutational gains within the antigen itself (9). In addition to antigen presentation, T-cell infiltration is vital for therapeutic success. It is known that solid tumors have the capacity to limit T-cell infiltration through secretion of immunosuppressive cytokines, disruption of T-cell homing, and promoting abnormal vasculature (10). T-cells must also combat with the progressive loss of T cell effector functions known as T cell exhaustion. This is due primarily to the constant presence of antigen and inflammation, which leads to an upregulation of immunosuppressive receptors such as CTLA-4 and PD-1 (11). Immune checkpoint blockade can help alleviate immunosuppression that occurs during exhaustion, but the underlying mechanisms for exhaustion are still not fully understood making it a challenge to reinvigorate T-cells from this state.

### Tumor Microenvironment and Immunotherapy

Reprogramming the TME is seen as a viable option to overcome the challenges that exist for immunotherapy in solid tumors. Normalizing tumor vasculature has the potential to reverse immune evasion and increase the infiltration of immune cells. Normalizing vasculature can be done using antiangiogenic agents (12–14). It has also been hypothesized that this could be achieved through the alternative approach of vascular detransformation, a method that would look to reverse endothelial-mesenchymal transition (Endo-MT) that occurs within the TME (15). These transformed endothelial cells have been shown in glioma to acquire a mesenchymal phenotype that leads to vessel sprouting and outgrowth making them a novel target (16, 17). Additionally, myeloid cells, specifically tumor-associated macrophages (TAMs), are also a prime target to reprogram the TME due to their ability promote angiogenesis, metastasis, and immunosuppression across various cancer types (12, 18). TAMs can be targeted using selective inhibitors of colony-stimulating factor 1 receptor (CSF1R) and PI3K kinase, neutralizing antibodies of CSF-1, Toll-like receptor (TLR) agonists, and DNA binding agents that selectively induce cell cycle arrest in monocytes and macrophages (18–21). Another group of immunosuppressive cells within the TME, known as regulatory T-cells (T_reg_), have been shown to be recruited by chemokines produced by tumor cells, cancer-associated fibroblasts, and an immunosuppressive subset of TAMs (22, 23). T_regs_ are normally required to balance T-cell effector function, however they are recruited to suppress the antitumor immune response and promote tumor progression within the TME (12, 24). Outright T_reg_ ablation is not seen as a viable option to improve immunotherapy as the absence of T_regs_ would increase the occurrence of adverse events due to overactive T cell effector function (25). The reprograming of T_regs_ could be achieved through metabolic manipulation *via* protein kinase B (AKT) and through downregulation of transcription factors such as Helios, NF-kB, Eos, Bach2, and Nr4a (26). It has been found that once T_regs_ have been reprogrammed they will begin to exhibit a decrease in Foxp3 expression, gain immune-stimulating function, and develop a T helper cell phenotype (26).

## Vascular Microenvironment and Tumor Immunity

### Mechanisms Underlying Vascular Regulation of Tumor Immunity

**Figure 1:**
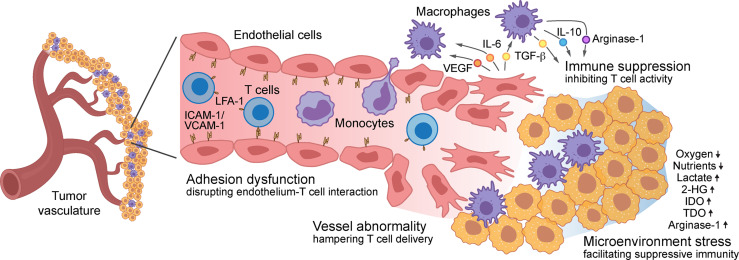
Role of vascular microenvironment in regulation of tumor immunity – Vascular microenvironment regulates T cell-based tumor immunity *via* multifactorial mechanisms: structurally and topologically abnormal vasculature hampers T cell infiltration; downregulated expression of adhesion molecules, such as ICAM-1 and VCAM-1 in tumor-associated endothelial cells (ECs), impedes T cell adhesion to and diapedesis through the tumor vessel wall; tumor ECs produce multiple cytokines that induce immunosuppressive phenotypes in macrophages and inhibit T cell survival and activation; the vascular microenvironment generates locoregional stresses including hypoxia, nutrient deprivation, and excessive immunosuppressive metabolites and enzyme, reducing T cell activity and inducing T cell exhaustion.

Aberrant vascularity characterizes malignant solid tumors, fueling the formation of an immune-hostile TME and inducing tumor resistance to immunotherapy, which involves multifactorial mechanisms: tumor vasculature becomes structurally and topologically abnormal, hampering the infiltration and therapeutic delivery of T cells into the tumors; expression of adhesion proteins including ICAM-1 and VCAM-1 are down-regulated in tumor-associated ECs, impeding T cell adhesion to and diapedesis through the tumor vessel wall; tumor ECs form a vascular niche and produce multiple growth factors and cytokines that induce immunosuppressive phenotypes in TAMs and inhibit survival and activation in T cells; tumor capillaries with functional abnormalities generate a vascular microenvironment with locoregional stresses including heterogeneous hypoxia and nutrient deprivation, reducing T cell activity and inducing T cell exhaustion. All of these mechanisms facilitate pro-tumor immunity and collectively lead to tumor evasion of immune responses and resistance to immunotherapies (**Figure 1**).

#### Vessel Abnormality

ECs have specialized roles in regulating many essential functions in the body that differs from tissue to tissue (27). However, a constant remains where endothelial cells throughout the body mediate the flow of blood, regulate the supply of nutrients, oxygen, and other solutes, and manage the migration of immune cells into and out of tissues. Vasculature begins to change when in the presence of a tumor, where these tumor ECs exhibit a defective endothelial monolayer, large intracellular openings and holes and abnormal sprouts (28, 29). The changes that occur within the vasculature could be initially driven by chronic tumor-expressed vascular endothelial growth factor (VEGF) stimulation (30), followed by EC plasticity-mediated permanent phenotypic changes (15). In addition, pericytes are vital cells that play an important role in vessel maintenance and remodeling; however, in tumor vasculature they are either absent or exhibit abnormal function leaving vessels to become leaky. The leakiness of the tumor vasculature results in irregular blood flow and ultimately dysfunctional trafficking of lymphocytes (31). These structural changes lead to vessel abnormalities and inadequate perfusion resulting in insufficient delivery of therapeutic agents and lymphocytes.

#### Microenvironmental Stress

Structural and topological vessel aberrancy causes regions of hypoxia and nutrient depletion within the tumors (29). Due to cancer’s intensification of metabolic demands, there is less blood oxygenation which means the vascular density does not need to be lower than normal tissue for hypoxia to occur (32). Hypoxia inducible factor 1-alpha (HIF1α), the master regulator of hypoxia, further drives upregulation of VEGF which has been shown to enhance tumor survivability through activation of the MAPK/ERK pathway (33). Additionally, as cancer consumes glucose within the TME, it secretes lactate creating an acidic environment that aids in the stabilization of HIF-1α mRNA (34). The lactate produced by the cancer cells further drives angiogenesis in tumor ECs *via* the lactate importer monocarboxylate transporter 1 (MCT1) which activates NF-κB and HIF-1α (34). Further crosstalk between tumor cells and ECs can influence the ECs to adopt a prothrombotic, proinflammatory, and cell-adhesive state known as EC dysfunctional activation (35). These “activated” ECs can promote aggressive tumor cell phenotypes that are drug resistant and have an increased risk of metastasizing increasing the difficulty of implementing a wide range of treatments (36, 37), and also facilitate pro-tumor immunity.

#### Immunosuppressive Niche

Tumor ECs produce a wide range of angiocrine factors that can promote angiogenesis, tumorigenesis, chemoresistance, and immune suppression (38–45). One such angiocrine, interleukin-6 (IL-6), has been shown to promote alternative TAM polarization *via* induction of HIF-2α in glioblastoma (45), leading to TAM secretion of immunosuppressive molecules, such as TGF-β, IL-10, and Argainse-1, that inhibit T cell activation. Tumor ECs also recruit TAMs through the production of CCL2, M-CSF, and VEGF which upon entry into the TME promote angiogenesis, immune suppression, and metastasis (28, 46–48). This mechanism demonstrates how tumor ECs can promote tumor progression through immunosuppression, thus further highlighting the importance of cross talk between different cells within the TME.

#### Adhesion Dysfunction

Adding on to the difficulties of treating solid tumors is the interaction or lack thereof, that takes place between tumor ECs and lymphocytes. Not only can tumor ECs activate or inactivate the immune cells within the TME, but they may selectively recruit immune cells to promote tumorigenesis and immune evasion. Interactions between leukocytes and ECs are mediated by expression of adhesion molecules on the cell surface of ECs, which mainly include intercellular adhesion molecule-1 (ICAM-1), vascular cell adhesion molecule-1 (VCAM-1), E-selectin, P-selectin, platelet–endothelial-cell adhesion molecule-1 (PECAM-1, CD31), and CD99 (49, 50). Infiltration of immune cells into the tumor is a multistep event subjected to temporospatial regulation of lymphocyte adhesion to, rolling at, and transmigration across the tumor ECs. Exposure of ECs to proinflammatory stimulus with LPS, TNF-α, or IL-1β upregulates ICAM-1 and VCAM-1 expression, as a natural process for lymphocyte recruitment and infiltration (50, 51); however, dysfunctional expression of adhesion molecules, such as ICAM-1, reduces T cell adhesion to tumor endothelium, likely due to microenvironmental cues that reprogrammed ECs, making it harder to mount an anti-tumor immune response (47, 52, 53). Adhesion molecules linked to leukocyte binding are suppressed by angiogenic factors leading tumor ECs to adopt a state known as endothelial anergy where there is a lack of an immune response to the presence of proinflammatory stimuli such as IL-1, TNF-α, and IFNγ (47, 53–55). Additionally, tumor cells upregulate the ligand enothelin-1 in ovarian cancer, which in turn binds to endothelin B receptor on tumor ECs, resulting in the inhibition of ICAM1 expression thus preventing lymphocyte infiltration (56). Alongside this unresponsive state, tumor ECs themselves have shown to downregulate adhesion molecules and chemokines. The tumor vascular microenvironment creates problems for all solid tumor treatment options, and it’s important to understand the underlying mechanisms in order to remodel the TME to increase the efficacy of therapeutics.

### Immune Cell Infiltration

It is well documented that the presence of preexisting tumor-infiltrating lymphocytes (TILs), which include T cells, B cells, and natural killer (NK) cells, usually indicates a positive response to treatment (57, 58). Tumors can combat TILs by disrupting lymphocyte homing and infiltration. Trafficking of lymphocytes is regulated through chemokines and adhesion molecules. Chemokines mediate chemotaxis through gradients and initiate the active form of ligands expressed on the surface of lymphocytes allowing them to transmigrate into the tissue. Tumors have been shown to hinder lymphocyte chemotaxis by suppressing expression of chemokines that promote T-cell infiltration such as CCL4 and CCL27 in melanoma (10, 59). Lymphocyte homing and infiltration can also be altered by posttranslational nitrosylation of CCL2 to nullify its ability to recruit tumor specific cytotoxic T lymphocytes (CTLs) making it hard to implement immunotherapy using antigen specific T cells (60). Aberrant vascularization presents a significant hindrance to lymphocyte infiltration, which inhibits T cell adhesion to tumor vasculature and hampers T cell delivery into the tumor. VEGF, a pro-angiogenic factor driving tumor angiogenesis and aberrant vascularity, likewise has also been shown to downregulate the expression of ligands required for T cell extravasation (10, 31).

### Immune Cell Function

The success of immunotherapies is not only dependent on the trafficking of lymphocytes, but also their function. T cells and NK cells require activation in order to produce their cytotoxic proteins, which tumors can evade through gene regulation and immune checkpoints. T cells require activation *via* their T cell receptor (TCR) that recognizes MHC class I molecules, conversely NK cells are inhibited by the presence of MHC class 1 molecules. MHC class I presentation is not enough to fully activate T cells; they must also receive signaling from adhesion and costimulatory molecules. It is common for tumor cells to lack these necessary components and instead express inhibitory ligands such as PD-L1 that will shut down T cell effector function (61, 62). Additionally, NK cells can be influenced within the TME to adopt a pro-angiogenic phenotype to promote tumor progression in non-small cell lung carcinomas (NSCLC) patients (63). Tumors also have the capability to downregulate MHC class I presentation, however, if the tumor cell is completely negative for MHC class I molecules then it will become susceptible to NK cells (64). In order to evade NK cells, cancer will maintain low expression of MHC class I molecules to prevent NK cells from initiating cell death, while simultaneously reducing the activation of T cells (65). In order for T cell and NK cell persistence to occur they must continually be exposed to stimulating cytokines such as IL-2 (62, 66). IL-2 has been used in clinical trials to help stimulate the anti-tumor response, but it is associated with dangerous toxicities and expansion of T_reg_ cells (62).

The components of the TME, including hypoxia, immunosuppressive cytokines and matrix, stroma cells, and anti-inflammatory leukocytes, propel T cells to adopt a less effective state known as exhaustion. Exhausted T cells exhibit a decrease in effector function and an increase in expression of inhibitory receptors such as PD-1 and CTLA-4 (67). Chronic exposure to tumor antigen combined with the presence of inhibitory ligands is thought to be the driving forces behind T cell exhaustion (68). Additionally, expression of TGF-β by tumor cells, fibroblasts, immune cells and tumor ECs quells the expression of cytotoxic T cell genes including perforin, granzymes and cytotoxins (69). The presence of other immune cells within the TME such as TAMs and myeloid-derived suppressor cells (MDSCs) negatively impact anti-tumor immune response. Macrophages play an important role in regulating immune response by adopting two different phenotypes: the pro-inflammatory M1 phenotype and the anti-inflammatory and immunosuppressive M2 phenotype. Upon entering the TME, most TAMs are polarized towards the M2 phenotype through signaling from tumor cells, T helper 2 (TH2) cells, Treg cells, and other cells or molecules in the TME (70). These M2 TAMs promote immunosuppression through expression of TGF-β and IL-10 thus being negatively correlated with therapeutic response (71, 72). Even though TAMs primarily adopt the M2 phenotype, polarization of macrophages is all about a balance of signals with their phenotype easily being swayed by changes in signaling opening the potential for macrophage reeducation. Immunosuppressive TAMs express PD-L1, the negative regulator of T and NK cells, where blocking of PD-L1 unlocks TAMs’ potential for anti-tumor activity, suggesting that immune checkpoint blockade could be a possible avenue for macrophage reeducation (70). Not only are TAMs immunosuppressive but they also stimulate angiogenesis through the production of pro-angiogenic factors such as VEGF, epidermal growth factor (EGF), basic fibroblast growth factor 2 (FGF2), IL-8, CXCL12, and TNFα further echoing the need for anti-TAM therapies (62). Similarly, MDSCs induce tumor immunosuppression with several additional mechanisms, such as the expression of PD-L1 and CD-80 to abrogate antigen-specific immune responses, and production of reactive oxygen species (ROS) which induces posttranslational modification of TCRs rendering them unresponsive to antigen presentation (73). MDSCs have also been implicated in promoting angiogenesis through increased production of fatty acid synthase which in turn activates PPARβ/δ-dependent expression of genes including VEGF (63). Pro-inflammatory neutrophils within the TME, whose primary role is to be the first line of defense against infection, have been negatively correlated with clinical outcome (63). These cells hold significant influence over tumor ECs through the wide range of secretory factors including IL-1β, VEGF, FGF2, TGFα, hepatocyte growth factor (HGF), and angiopoietin 1 (ANG1) (63).

ECs play a significant role in immune regulation. Tumor ECs may modify the expression of adhesion molecules to recruit specific types of immune cells, leading to favored recruitment of macrophages, T_reg_ cells, and MDSCs while inhibiting CD4^+^ and CD8^+^ T cells, NK cells, dendritic cells (DCs), and neutrophils (74). Tumor ECs lack the necessary stimulatory components needed to fully activate naïve T cells, and downregulate MHC associated genes to aid in immune evasion, as well as express inhibitory ligands such as PD-1 to further suppress T cell effector function (75). Additionally, tumor ECs can selectively induce T cell apoptosis through expression of FasL allowing them to eliminate CD8^+^ T cells while sparing T_reg_ cells (76). Crosstalk between tumor ECs and MDSCs creates a positive feedback loop involving VEGF: VEGF stimulates MDSC recruitment, while recruited MDSCs promote angiogenesis and immune suppression (77). Similarly, a feedback loop exists between TAMs and ECs within the hypoxic TME where tumor ECs express common lymphatic endothelial and vascular endothelial receptor (CLEVER1), which is an adhesion molecule that selectively recruits immunosuppressive cells (78).

### Immune Cell Metabolism

The TME is a battleground for metabolic resources with tumor cells, effector immune cells and immunosuppressive cells vying for limited nutrients essential for survival. Cancer cells increased metabolic demands combined with aberrant vasculature create an environment that is deficient of nutrients and hypoxic, greatly influencing what metabolic pathways cells within the TME can utilize (79). T cells switch from the mainly oxidative and fatty acid metabolism of naive and resting T cells to increased glucose uptake and glycolysis during activation. Metabolism of glucose is essential for CD4^+^ and CD8^+^ effector T cells; having received costimulatory signals these cells immediately begin to upregulate genes associated with glycolysis and the tricarboxylic acid cycle (TCA) to meet increased metabolic demands for proliferation (80). Additionally, the pentose phosphate pathway (PPP) is upregulated and provides the necessary NADPH required for fatty acid and plasma membrane synthesis (81). Nuclear factor of activated T cells (NFAT), which induces the vital stimulatory cytokine IL-2, requires ROS to be present in order for expression to occur making it strongly dependent upon the type of metabolism pathway utilized (79). Glucose deficiency in the TME causes the vital intermediate phosphoenolpyruvate, involved in NFAT expression, to become expressed thus losing IL-2 stimulation and T cell persistence (82). Mitochondrial respiration is essential for T cell effector function and consequently mitochondrial dysfunction has been identified as negatively correlated to treatment response when using CAR-T cells (83). It’s thought that a buildup of mitochondrial ROS levels causes mitochondrial dysfunction and has strongly been associated with the T cell exhaustion phenotype (79). Competition within the TME for crucial amino acids impacts the quality of the anti-tumor T cell response. Tumor cells, MDSCs, TAMs, and cancer associated fibroblasts (CAFs) actively deplete tryptophan through upregulation of indoleamine 2,3-dioxygenase (IDO), which its metabolite kynurenine has been shown to upregulate PD1 expression on CD8^+^ T cells (84, 85). Depletion of tryptophan is negatively correlated with patient outcome and further demonstrates the importance of metabolism to obtain a therapeutic immune response (86).

The suppressed blood circulation that exists in the vascular TME leads to locoregional accumulation of immune-hostile metabolites and enzymes including lactate, 2-hydroxyglutarate (2-HG), arginase-1, indoleamine 2,3-dioxygenase (IDO), and tryptophan 2,3-dioxygenase (TDO). For example, lactate can reach immunosuppressive concentrations of 20–30 mM in the vascular TME, as opposed to around 3 mM in normal tissues (87). Excess lactate and H^+^ suppress cell proliferation, survival, cytokine production and cytotoxicity in T and NK cells (88, 89). Oncometabolite 2-HG inhibits demethylases to increase histone methylation, and these epigenetic changes lead to suppressed T cell proliferation, TCR signaling, and NFAT activity (90). Arginase-1, derived from TAMs and MDSCs, depletes arginine, a critical amino acid for T cell activation (91). IDO and TDO, primarily produced by TAMs and CAFs, respectively, break down tryptophan and yield kynurenine, a suppressive metabolite that inhibits T cell functions (91–93). In addition, the aberrant vasculature of the TME contributes to promoting a hypoxic environment that inhibits effector T cell function, enhances Treg activity, and reduce NK cell cytotoxicity (94, 95). Hypoxia rewires T cell metabolism mainly *via* HIF-1α (96), but the precise *in vivo* role of HIF-1α in regulation of T cell function remains obscure (97): HIF-1α deficiency in CD8^+^ T cells enhances fatty acid catabolism and their anti-tumor functions (98), while HIF-1α promotes infiltration of CD8^+^ T cells into the tumors and enhances their effector responses to persistent antigen and promote tumor clearance (99, 100).

### Epigenetic Regulation of Immune Cells

Epigenetic regulation of immune cells, i.e., DNA methylation and histone modifications, determines their fate and activation. Understanding how these epigenetic changes impact immune cells within the TME is vital to reversal of tumor immunosuppression. As already noted, T cell exhaustion is a major problem for long term efficacy of immunotherapy. Exhausted T cells have been identified to have a unique chromatic architecture induced through epigenetic modifications (70, 101–103), implicating that epigenetic therapeutic agents could serve in adjuvant treatment for cancer immunotherapy. Likewise, PD1 blockade is a treatment option used to combat T cell exhaustion, however, this treatment does not fully restore effector T cell function suggesting the importance of the impact that epigenetic modifications have on T cell function (104). Recent studies identify HMG-box transcription factor TOX as a major regulator of genetic and epigenetic remodeling that occurs during T cell exhaustion (104–106). TOX is largely dispensable for the formation of T_effector_ and T_memory_ cells, but it is required for the development for T_exhaustion_ cells in chronic infection (105).

The epigenetic landscape in TAMs can be remodeled in response to acute stimulation and polarizing stimuli, which helps integrate signaling, such as NF-κB and STATs, over time and underlies reprogramming of TAMs to alter their gene expression (107). Most of the epigenetic research work has been focused on the macrophages with M1 phenotype, and the epigenetic modifiers involved in TAMs with M2 polarization remains largely unknown. Previous work shows that the histone demethylase JMJD3, induced by IL-4, promotes expression of M2-promoting transcription factor IRF4 by removing negative H3K27me3 marks at the Irf4 locus (70, 108, 109). Recent studies identify epigenetic enzymes, including DNMT1, PRMT6, and KDM6B, which regulate M2 polarization and tumor-promoting functions in TAMs (110, 111), serving as targets for disrupting TAM immunosuppressive phonotypes.

### Therapeutic Effects of Radiochemotherapy on Immune Cells

Chemotherapy and radiation have been two standard treatments against solid tumors; understanding how these cytotoxic therapies impact immune cells will better strategies of using synergistic therapies. It should be noted that not all cytotoxic strategies have a positive impact on immune cells. However, cytotoxic radiochemotherapies have emerged as a potential immune stimulant due to their involvement with immunogenic cell death (ICD) (112, 113). ICD is defined by a type of cancer cell death triggered by cytotoxic therapeutics, which activates long-lasting antitumor immunity; ICD proceeds by the release of damage-associated molecular patterns (DAMPs) that allow for the processing and presentation of tumor-associated antigens, in which priming T cells can turn the immune response from a tolerogenic one to an anti-tumor immunogenic response. In addition, ICD stimulates lymphocyte trafficking and infiltration, showing promise in improving TIL numbers in breast cancer, ovarian cancer and melanoma (114–116). Interestingly, there’s been evidence that certain cytotoxic chemotherapuetics can selectively target immune suppressive cells including T_reg_, MDSCs and TAMs (117–119). Dosage is crucial in eliciting these selective effects, maximum tolerated dose regimens have been shown to deplete CD8+ T cells and NK cells while low dose regimens have been shown to preferentially target MDSCs and T_reg_ cells (120–122). Better understanding of their therapeutic effects on immune cells will help design more effective immunotherapy. Similar to chemotherapy, radiation also elicits an immunogenic response *via* DAMPs (123). In addition, radiation can affect the tumor vasculature resulting in an upregulation of adhesion molecules, further stimulating the recruitment of T effector cells (124). Radiotherapy is one of the most widely used treatment options for solid tumors (113, 125). Recent studies show that radiation used in combination with immunotherapy, such as checkpoint blockade, elicit significant immune responses (126–128).

## Reprogram Vascular Microenvironment for Immunotherapy

### Anti-Angiogenic Therapy

**Figure 2:**
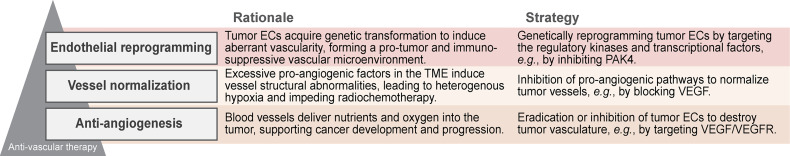
Strategies for anti-vascular therapy – Three therapeutic strategies have been developed for vasculature-targeting anti-cancer treatments, including anti-angiogenesis, vessel normalization, and endothelial programming.

Anti-vascular therapy was initially thought to be groundbreaking in cancer treatment (129). The concept of conventional anti-angiogenic therapy was to starve the tumor of oxygen and nutrients through eradication of its vasculature, primarily by inhibiting pro-angiogenesis factors and their downstream pathways such as VEGF (130, 131) (**Figure 2**). Ultimately, anti-angiogenic therapy exhibits small and transient benefits in most malignant cancers. These therapeutic difficulties and failures are due to multiple mechanisms that contributes to the tumor resistance to anti-VEGF treatment, including angiogenic pathway redundancy, compensatory activation of survival signals, and pericyte and macrophage-mediated protection (132). Furthermore, anti-angiogenic therapy-induced vascular shutdown can deteriorate tumor hypoxia, leading to more aggressive tumor phenotypes in tumor growth, invasion, and metastasis *via* HIF-1α; this also generates a hostile barrier for delivery of therapeutic agents and anti-tumor lymphocytes into the tumors. In addition, anti-angiogenesis has shown negative side effects including cardiac toxicity, hemorrhage, thrombosis and gastrointestinal perforation further making it hard to justify its use as a therapeutic (133–135).

### Vessel Normalization

Vessel normalization has emerged as a novel approach to combat aberrant tumor vasculature through restoration of vessel perfusion and oxygenation (136). The goal of this therapy is to structurally normalize tumor vasculature, leading to a decrease in intratumor hypoxia and an increase in the delivery of therapeutic drugs and the efficacy of radiotherapy (**Figure 2**). Based on the central hypothesis that vascular abnormalities are driven by imbalance of pro- and anti-angiogenic factors (29, 137), current vessel normalization therapies have focused on targeting excessive pro-angiogenic factors, such as VEGF and PlGF, using neutralizing antibodies and pharmacological inhibitors of their downstream tyrosine kinases. However, these therapies have shown transient effects on tumor oxygenation and small benefits (138–146). Ideally, these vessel-normalizing treatment can reduce intratumoral hypoxia and enhance delivery of immunotherapeutic agents or cells into the TME, providing certain opportunities for improving immunotherapy, which needs further optimization of the therapeutic dose and duration to reach maximal and persistent effects on vessel normalization.

Another therapeutic field of vessel normalization is vascular maturation, as the tumor vasculature is characterized by disrupted coverage of pericytes that stabilize vascular structure and maintain its functional integrity (137). VEGF2 blockade can recruit pericytes through activation of Ang1 and Tie2 signaling (138). The risk of hemorrhage or venous thromboembolism exists whenever targeting VEGF, which has led to research into other targets to normalize vasculature. Targeting the upregulation of PDGF-β is seen as another option as it has been reported in mouse models to increase pericyte recruitment while decreasing EC proliferation (147, 148). However, there has been instances reported where upregulation of PDGF-β led to tumor growth (17).

### Endothelial Reprogramming

As an alternative process to the vascular abnormality mechanism driven by angiogenic factor-mediated vessel sprouting and outgrowth, tumor ECs undergo genetic programming to induce aberrant vascularity. Robust cell plasticity in ECs has been well characterized in embryonic development (149–153). ECs undergo endothelial mesenchymal transition (Endo-MT) to *de novo* generate fibroblasts, stem-like cells, and smooth muscle cells in pathological settings including cardiac, renal and liver fibrosis, ossifying myositis, vascular inflammation, and cerebral cavernous malformation (154–160). Our recent work reveals that tumor ECs retain key endothelial functions but acquire mesenchymal phenotypes including enhanced proliferative and migratory capacities *via* cell transformation, i.e., partial Endo-MT, therefore driving aberrant vasculature in TME (16, 161, 162); this cell plasticity-mediated mechanism provides a new insight into vascular abnormality in TME, suggesting vascular de-transformation as a new strategy for cancer therapy (15) (**Figure 2**). Theoretically, tumor ECs are driven by plasticity-mediated genetic reprogramming where the hope of reversing this would offer a non-transient effect on reforming vessel morphology in the TME. The strategy for EC reprogramming focuses on the key regulatory node that drives the abnormal structure features. Our recent kinome-wide genetic screening of mesenchymal-like transcriptional activation in tumor ECs identifies PAK4 as an innovative target to reprogram ECs in glioblastoma (163). Genetic ablation or pharmacological inhibition of PAK4 showed an increase in expression of adhesion molecules in tumor ECs, a decrease in vessel abnormalities with improved T-cell infiltration, rendering tumors more sensitive to CAR T immunotherapy (163).

Additional benefits for endothelial reprogramming include the potential effects on anti-tumor immunity *via* improving vessel perfusion to remove immunosuppressive metabolites and enzymes in the TME as well as *via* directly reconditioning immunosuppressive vascular niche. Our recent work reveals that transformed ECs in TMEs form an immunosuppressive vascular niche *via* producing IL-6 that induces M2 phenotypes in TAMs, which inhibits T cell infiltration into and activation at the TME (45, 164); endothelial reprogramming may, therefore, generate a locoregional host-friendly TME with anti-tumor immunity that allows a successful immunotherapy. These findings offer proof of concept that reprograming tumor ECs is a viable option to reverse immune suppression within the TME and improve the efficacy of T cell-based immunotherapy.

In summary, we overview the role of the vascular TME in tumor evasion of immune responses and resistance to immunotherapy, with a focus on vessel abnormality, dysfunctional adhesion, immunosuppressive niche, and microenvironmental stress (**Figure 1**). We propose that development of new therapeutic approaches in order to reprogram tumor ECs may offer exciting opportunities to recondition the TME and to overcome tumor resistance to T cell-based immunotherapy (**Figure 2**).

## Author Contributions

ZL wrote the manuscript. YF supervised the work. All authors contributed to the article and approved the submitted version.

## Funding

This work was supported in part by National Institutes of Health (NIH) grants T32GM008076 (to ZL) and R01NS094533, R01NS106108 and R01CA241501 (to YF). The content is solely the responsibility of the authors and does not necessarily represent the official views of the NIH.

## Conflict of Interest

The authors declare that the research was conducted in the absence of any commercial or financial relationships that could be construed as a potential conflict of interest.

## Publisher’s Note

All claims expressed in this article are solely those of the authors and do not necessarily represent those of their affiliated organizations, or those of the publisher, the editors and the reviewers. Any product that may be evaluated in this article, or claim that may be made by its manufacturer, is not guaranteed or endorsed by the publisher.
